# The Association Between Metabolomic and Usual Biochemical Data Helps to Detect Insulin Resistance

**DOI:** 10.3390/biomedicines14020393

**Published:** 2026-02-09

**Authors:** Fábio S. Pimenta, Camila Conde, Radael R. Rodrigues Júnior, Bianca P. Campagnaro, Thiago M. C. Pereira, Manuel Campos-Toimil, Silvana S. Meyrelles, Elisardo C. Vasquez

**Affiliations:** 1 Pharmaceutical Sciences Graduate Program, Vila Velha University (UVV), Vila Velha 29102-920, ES, Brazil; drfabiopimenta@gmail.com (F.S.P.);; 2 Centro de Investigación en Medicina Molecular y Enfermedades Crónicas (CIMUS), Universidade de Santiago de Compostela, 15782 Santiago de Compostela, Spain; 3 Graduate Program in Physiological Sciences, Federal University of Espírito Santo (UFES), Vitória 29047-105, ES, Brazil; silvana.meyrelles@ufes.br

**Keywords:** insulin resistance, metabolomics, organic acids, primary prevention, immunometabolism

## Abstract

**Background**: Chronic noncommunicable diseases account for nearly 80% of global deaths and are strongly associated with insulin resistance (IR). One of the most significant clinical findings of the past two decades is that the molecular mechanisms underlying immune and metabolic systems have been evolutionarily conserved across species. **Methods**: This study included 34 volunteers (19 men and 15 women). Demographic data were collected using validated questionnaires. Anthropometric measurements (weight, height, waist-to-hip ratio, and body composition assessed by tetrapolar bioimpedance) were obtained directly. Laboratory analyses included fasting glucose and insulin, glycated hemoglobin, HDL cholesterol, total cholesterol, triglycerides, organic aciduria, and additional biochemical markers assessed using standard methods. Group comparisons were performed using parametric or nonparametric statistical tests according to data distribution, as specified in the figure legends. **Results**: The primary analyses focused on identifying early metabolomic alterations associated with insulin resistance in individuals whose conventional biochemical parameters were within laboratory reference ranges. Individuals with a TG/HDL ratio > 2 and increased urinary kynurenate excretion exhibited a 3.6-fold higher relative risk of insulin resistance, while elevated insulin levels combined with urinary α-ketoisovalerate were associated with a 2.7-fold increased risk. Significant differences in plasma insulin, HbA1c, and HOMA-IR were observed between healthy and diseased individuals (*p* < 0.05), indicating early metabolic dysfunction preceding clinical disease onset. **Conclusions**: Metabolomic biomarkers serve as reliable indicators of subclinical metabolic disturbances, revealing significant risks in major metabolic pathways even in individuals with conventional exams within normal limits. Early detection through these metabolomic markers may enable personalized interventions aimed at preserving cellular function and systemic metabolic balance.

## 1. Introduction

Insulin resistance (IR) is a pathophysiological condition characterized by reduced sensitivity of target tissues to normal or elevated circulating levels of insulin. Together with chronic low-grade inflammation, IR is involved in a wide range of immunometabolic disorders that impose a growing economic burden on global health systems. These conditions are closely associated with comorbidities such as type 2 diabetes mellitus (T2DM), obesity, non-alcoholic fatty liver disease (NAFLD), cardiovascular diseases (CVD), and accelerated aging. Collectively, these disorders play a direct or indirect role in chronic non-communicable diseases, which account for the majority of deaths worldwide [1,2,3,4,5,6] and are also a central focus of our research group [7,8,9].

Currently, substantial evidence demonstrates that inflammation is an evolutionarily conserved process that is biochemically integrated with the metabolic system, representing the result of approximately 600 million to 1 billion years of adaptive evolution [3,10,11,12]. The diseases responsible for most deaths worldwide arise from the convergence of immunometabolic conditions in which chronic low-grade inflammation plays a central role in their pathophysiology through an intricate network of cross-communication pathways.

Universally used laboratory reference values assist health professionals in interpreting results for diagnostic formulation and clinical decision-making [13]. However, the research underlying these reference values is rarely specified by clinical laboratories [14,15]. Estimates of reference ranges are generally derived from studies based on random samples of specific populations [16], predominantly conducted in developed countries, whose values are then adopted as global reference standards.

The relationship between plasma triacylglycerol and high-density lipoprotein cholesterol levels (TG/HDL-C) has proven useful in predicting the occurrence of obesity, hypertension, and type 2 diabetes mellitus (T2DM), as well as in preventing cardiovascular events. This topic has been extensively investigated by our research group using both animal disease models [9] and clinical studies [17].

However, although appropriate cutoff values for this purpose have been proposed [18,19], further validation and confirmation using additional analytical tools are still required. Conventional biochemical markers often fail to detect early metabolic dysfunction, highlighting the need for more sensitive approaches capable of identifying subclinical immunometabolic alterations. The catabolic pathway of L-tryptophan (TRP), an essential amino acid—also known as the kynurenine pathway (KP)—produces several intermediate metabolites with distinct metabolic functions, including kynurenine (KYN). This pathway operates primarily in the liver, intestine, kidney, and other tissues. Evidence indicates that the enzyme tryptophan 2,3-dioxygenase (TDO) regulates TRP degradation under basal conditions, whereas indoleamine 2,3-dioxygenase (IDO) is induced and modulated by inflammatory stimuli [20]. α-Ketoisovalerate is an organic acid derived from the catabolism of the branched-chain amino acid (BCAA) L-valine, which undergoes transamination catalyzed by branched-chain amino acid transaminase (BCAT).

The first two steps of branched-chain amino acid (BCAA) catabolism are shared among all three BCAAs. The initial step is transamination, catalyzed by branched-chain amino acid transaminase (BCAT), followed by oxidative decarboxylation catalyzed by the branched-chain α-ketoacid dehydrogenase (BCKDH) complex. Notably, the transamination reactions are reversible and generate branched-chain α-keto acids—α-ketoisocaproate, α-keto-β-methylvalerate, and α-ketoisovalerate—from L-leucine, L-isoleucine, and L-valine, respectively. The BCKDH-catalyzed reaction represents the rate-limiting step in BCAA catabolism and is therefore tightly regulated. Its activity depends on several cofactors, including thiamine, riboflavin, niacinamide, pantothenate, α-lipoic acid, and the mineral magnesium [21]. Impaired BCAA catabolism may lead to elevated circulating BCAA levels, which have been associated with insulin resistance (IR), type 2 diabetes mellitus (T2DM), and cardiovascular diseases (CVD) [22,23]. This study included volunteer participants recruited through convenience sampling, comprising healthy, overweight, and obese individuals, with and without type 2 diabetes mellitus (T2DM). Although the overall cohort included participants with diagnosed metabolic conditions, analyses relevant to the study objective were focused on individuals whose conventional biochemical parameters fell within normal laboratory reference values, in whom metabolomic alterations were specifically investigated. The aim of this clinical study was to identify alterations in metabolic pathways associated with IR and chronic low-grade inflammation that compromise the immunometabolic system in a cohort of volunteer participants presenting biochemical test results within normal laboratory reference ranges.

## 2. Materials and Methods

This study was conducted in accordance with the Ethical Principles for Medical Research Involving Human Subjects outlined in the 1975 Declaration of Helsinki and was approved by the Ethics Committee of Vila Velha University (protocol number 3,769,978). All participants provided written informed consent prior to enrollment. A total of 34 volunteer participants were recruited by convenience sampling at our outpatient clinic (Clínica Dr. Fábio Pimenta, Brazil). Participants were aged between 31 and 75 years and were included without discrimination regarding sex or ethnicity. Blood pressure was measured using a noninvasive method in accordance with the guidelines of international hypertension societies. Measurements were performed by a single trained evaluator using a manual sphygmomanometer (Welch Allyn DS44-11; Welch Allyn, Skaneateles Falls, NY, USA). Blood pressure was assessed in different orthostatic positions, and all procedures strictly followed international recommendations. Height was measured using a stadiometer (Seca 2013; Seca, Hamburg, Germany), and body weight was measured using a digital scale (Omron HBF-510LA; Omron Healthcare Inc., Lake Forest, IL, USA) with an accuracy of 0.1 kg. Body mass index (BMI) was calculated as weight (kg) divided by height squared (m^2^). Waist circumference was measured using a fiberglass anthropometric tape at the midpoint between the lower margin of the last rib and the iliac crest, with the participant standing and the tape positioned horizontally. Participants were classified according to BMI as follows: eutrophic (18.5–24.9 kg/m^2^), overweight (25.0–29.9 kg/m^2^), and obese (≥30.0 kg/m^2^). Insulin resistance was estimated using the Homeostasis Model Assessment of Insulin Resistance (HOMA-IR), calculated according to the internationally validated formula [24]: HOMA-IR = fasting insulin (µU/mL) × fasting glucose (mmol/L)/22.5, where fasting insulin (FI) and fasting glucose (FG) were measured after an overnight fast. Non-diabetic volunteers using medications known to interfere with insulin sensitivity (e.g., corticosteroids, antipsychotics, or antidepressants) were excluded from the study. Participants with a confirmed diagnosis of T2DM were included and maintained on their standard therapeutic regimen, which consisted of oral metformin (500 mg to 1 g/day) and/or insulin therapy. Systemic arterial hypertension (SAH) was defined as systolic blood pressure ≥ 140 mmHg and/or diastolic blood pressure ≥ 90 mmHg, or current use of any antihypertensive medication. Hyperlipidemia was defined as low-density lipoprotein cholesterol ≥ 160 mg/dL (≥4.1 mmol/L) or triglyceride levels ≥ 150 mg/dL [25].

### 2.1. Analyses Performed

The study was designed using convenience sampling and included 34 participants (19 men and 15 women). Of these, 17 participants were classified as obese and/or diagnosed with type 2 diabetes mellitus (T2DM), while the remaining 17 were normal-weight and/or overweight individuals. Demographic data were collected using validated questionnaires. Anthropometric measurements were obtained directly and included body weight, height, waist-to-hip ratio, and body composition assessed by tetrapolar bioimpedance. Laboratory biochemical analyses comprised fasting blood glucose and insulin, glycated hemoglobin (HbA1c), high-density lipoprotein cholesterol (HDL-C), total cholesterol, and triglycerides. Additional biomarkers (fibrinogen, apolipoproteins, homocysteine, γ-GT, hs-CRP, lipoprotein(a), urea, and creatinine) were assessed for clinical characterization but are not presented, as no significant associations with insulin resistance were observed. In addition, urinary organic acid profiles (organic aciduria) were assessed. For the specific analyses addressing the primary study objective (early detection), participants were stratified based on their biochemical profile. The ‘normometabolic’ subgroup included all individuals—regardless of BMI or clinical history—whose conventional biochemical parameters (fasting glucose and lipid profile) fell within standard laboratory reference ranges.

#### Participants and Baseline Characteristics

Baseline demographic, behavioral, and socioeconomic characteristics were recorded for all participants. The study population consisted exclusively of non-smokers, and alcohol consumption was limited to occasional or social use, with no reported history of alcohol abuse. All participants had completed secondary or higher education. Regarding lifestyle patterns, individuals classified as normal weight or overweight were predominantly physically active. In contrast, participants with obesity and/or diabetes exhibited predominantly sedentary behavior. These lifestyle variables were strictly considered during the interpretation of the metabolic analyses.

### 2.2. Anthropometric Measurements

Body composition was assessed by direct segmental multifrequency bioelectrical impedance analysis (InBody^®^), using a tetrapolar system with 15 impedance measurements obtained at three different frequencies (5 kHz, 50 kHz, and 250 kHz) across five body segments (right arm, left arm, trunk, right leg, and left leg). All measurements were performed at the Institute of Advanced Nutrition and Metabolism, Brazil. Anthropometric measurements were recorded in centimeters. Obesity was defined using a combination of body mass index (BMI) and waist circumference (WC), in accordance with clinical guidelines established by the National Institutes of Health (NIH) [26]. Participants were classified as obese if BMI was ≥30 kg/m^2^. Individuals with a BMI between 25.0 and 29.9 kg/m^2^ were classified as high-risk overweight (obesity equivalent) when WC exceeded 102 cm for men or 88 cm for women. Metabolic pathways were evaluated through the analysis of urinary organic acids (organic aciduria) using liquid chromatography coupled with tandem mass spectrometric detection (LC–MS/MS), as previously described [27,28]. Each participant received a pre-labeled insulated container provided by the partner laboratory, which included a protocol form for recording personal and sample-related information, a urine collection bottle, a test tube, a dropper for sample transfer, and a pre-addressed and labeled transport bag. Participants were instructed to collect their first morning urine sample upon waking. Subsequently, 9–10 mL of urine was transferred to the test tube using the provided pipette. The samples were stored in a domestic freezer to preserve integrity until delivery and, after a minimum of 6 h, were transported to the researcher for analysis.

### 2.3. Biostatistical Analysis

Data were expressed as mean or median, with variability reported as standard deviation (SD), standard error of the mean (SEM), and percentiles (P25, P50, and P75). Data distribution was initially assessed for normality using the D’Agostino–Pearson test. Although most parameters exhibited a Gaussian distribution, nonparametric graphical representations using percentile values were also included for selected variables that did not fully meet normality assumptions, particularly urinary organic acids. Accordingly, nonparametric statistical tests were applied when appropriate, as specified in the figure legends. When normality was confirmed, parametric statistical tests were performed. Student’s *t* test for paired samples was used when two measurements from the same individual at different time points were compared, while the independent-samples *t* test was applied for comparisons between healthy individuals and those with metabolic diseases.

One-, two-, or three-way analysis of variance (ANOVA) was performed depending on the number of independent variables analyzed, including treatment (one-way), treatment and sex (two-way), or treatment, sex, and age (three-way). When ANOVA results were significant, post hoc tests were applied to identify group differences. Statistical significance was defined as *p* < 0.05. Associations between categorical variables and the calculation of relative risk (RR) were evaluated using the chi-square test or Fisher’s exact test, when appropriate. All statistical analyses were performed using GraphPad Prism software (version 10.0). The specific statistical tests applied are described in the corresponding figure legends.

## 3. Results

### Anthropometric Analysis

**Figure 1:**
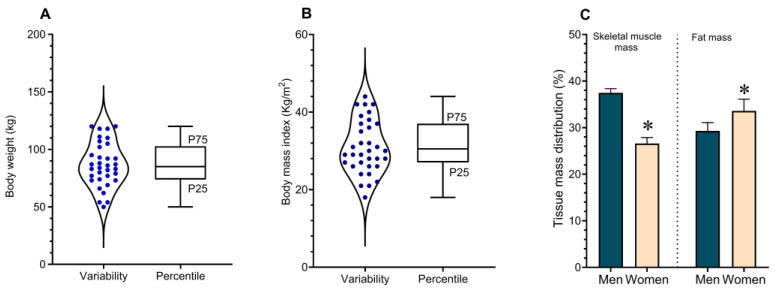
Anthropometric characteristics of the study population. (**A**,**B**) show individual variability of body weight and body mass index (BMI), respectively, represented by violin plots. Box-and-whisker plots indicate the 25th, 50th (median), and 75th percentiles. (**C**) Comparison of body composition between men and women, including skeletal muscle mass and body fat percentage. Statistically significant differences between sexes are indicated by an asterisk (*p* = 0.003; Student’s *t* test for independent samples).

Body weight in the study population ranged from 50 to 120 kg, with a mean value of 87 ± 3 kg (Figure 1A). Body mass index (BMI) ranged from 19 to 42 kg/m^2^, with a mean of 30 ± 1.1 kg/m^2^ (Figure 1B). The mean body weight of men was 91 ± 20 kg, with a mean BMI of 30 ± 6 kg/m^2^. In women, mean body weight was 73 ± 20 kg and mean BMI was 28 ± 6 kg/m^2^. These differences between sexes were statistically significant (*p* < 0.003). Body fat percentage was higher in women (34 ± 2.5%) compared with men (29 ± 1.8%). Conversely, skeletal muscle mass was significantly greater in men (37 ± 1.0 kg) than in women (26 ± 1.3 kg), with a mean difference exceeding 11 kg (Figure 1C). In contrast to skeletal muscle mass, fat mass was significantly higher in women, indicating greater adiposity in this group.

As shown in Table 1, adipose mass was significantly increased in both men and women classified as obese or diabetic compared with their respective normal-weight groups, indicating a marked increase in adiposity. Sex-related differences in body weight and fat mass were also observed across categories. It is noteworthy that resting metabolic rate is directly proportional to fat-free mass, which may partially explain the observed differences in body composition between men and women.

**Figure 2:**
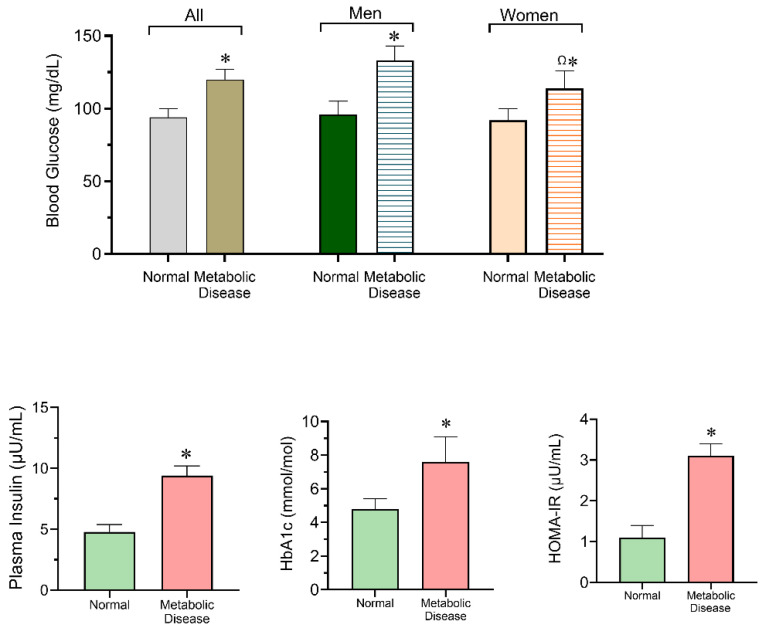
Metabolic and insulin resistance-related parameters in the study population. Mean fasting blood glucose levels comparing healthy individuals and those with metabolic diseases. The left panel shows the total sample, while the middle and right panels show comparisons stratified by sex (men and women). Plasma insulin levels, glycated hemoglobin (HbA1c), and Homeostasis Model Assessment of Insulin Resistance (HOMA-IR) comparing healthy individuals with metabolic disease patients. Statistical comparisons were performed using the independent-samples *t*-test. Values are expressed as mean ± standard deviation (SD). * *p* < 0.05 compared with healthy individuals. Ω indicates *p* < 0.05 compared with men with metabolic disease.

As shown in Figure 2, fasting blood glucose levels were significantly higher in individuals with metabolic disease compared with healthy participants in the total sample (*p* < 0.05). When stratified by sex, both men and women with metabolic disease exhibited significantly elevated glucose levels compared with their healthy counterparts, with women showing higher glucose levels than diseased men (Ω, *p* < 0.05). Plasma insulin levels were significantly increased in individuals with metabolic disease compared with healthy individuals. Similarly, glycated hemoglobin (HbA1c) levels were significantly higher in the metabolic disease group. These alterations were accompanied by a marked increase in HOMA-IR values in individuals with metabolic disease, indicating impaired insulin sensitivity.

**Figure 3:**
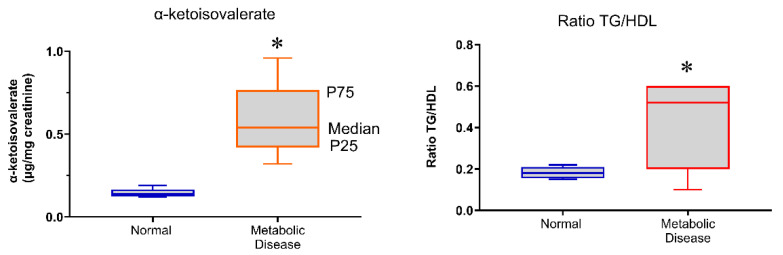
Urinary organic acid and lipid ratio alterations in the study population. The left panel shows urinary α-ketoisovalerate concentrations, and the right panel shows the triglyceride-to-HDL cholesterol (TG/HDL-C) ratio in metabolically healthy individuals and those with metabolic disease. Statistical comparisons were performed using the Mann–Whitney U test. Boxplots represent the 25th, 50th (median), and 75th percentiles. * *p* < 0.05 compared with healthy individuals.

As shown in Figure 3, individuals with metabolic disease exhibited significantly higher urinary α-ketoisovalerate levels compared with metabolically healthy volunteers (*p* < 0.05). In parallel, the triglyceride-to-HDL cholesterol ratio (TG/HDL-C) was also significantly increased in the metabolic disease group relative to healthy individuals (*p* < 0.05).

These findings indicate alterations in branched-chain amino acid (BCAA) catabolism and lipid metabolism in individuals with metabolic disease, even before overt clinical decompensation.

Given the multifactorial nature of metabolic diseases and the potential interactions among metabolic alterations, relative risk (RR) was calculated based on insulin levels in association with urinary organic acid excretion. Volunteers presenting a triglyceride-to-HDL cholesterol ratio (TG/HDL) greater than 2 in combination with increased urinary kynurenic acid excretion exhibited a 3.6-fold higher estimated risk of insulin resistance, based on cross-sectional categorical analysis. Additionally, individuals with elevated plasma insulin levels and urinary excretion of α-ketoisovalerate demonstrated a 2.7-fold higher estimated risk of insulin resistance.

**Figure 4:**
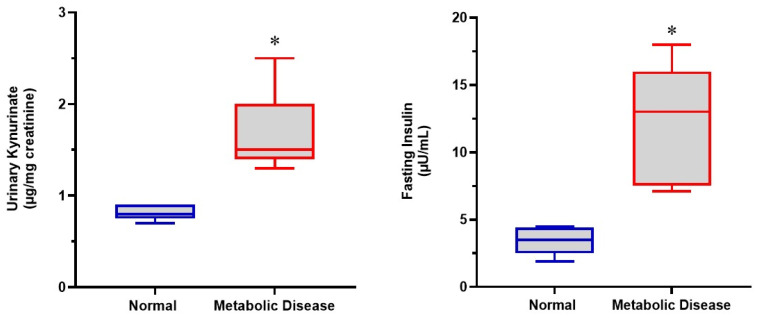
Urinary kynurenate excretion (µg/mg creatinine) and fasting insulin levels (µIU/mL) in metabolically healthy volunteers and individuals with metabolic disease. Boxplots represent the 25th, 50th (median), and 75th percentiles. * *p* < 0.05 compared with healthy individuals. Statistical comparisons were performed using the Mann–Whitney U test.

Figure 4 illustrates the comparison of urinary kynurenate excretion and fasting insulin levels between metabolically healthy volunteers and individuals with metabolic disease. Individuals with metabolic disease exhibited significantly higher urinary kynurenate levels compared with healthy controls, indicating alterations in tryptophan catabolism via activation of the kynurenine pathway. In parallel, fasting insulin levels were markedly increased in individuals with metabolic disease, reflecting impaired insulin sensitivity.

## 4. Discussion

The present study investigated early metabolic alterations associated with insulin resistance (IR) using urinary organic acid profiling in a convenience sample encompassing different body composition phenotypes. The main findings demonstrate that elevations in urinary α-ketoisovalerate and kynurenate are associated with fasting plasma insulin levels above 6 µU/mL and a triglyceride-to-HDL cholesterol ratio greater than 2, respectively. Importantly, these biochemical alterations were detectable even in obese individuals whose conventional laboratory parameters remained within reference ranges. This reinforces the potential of metabolomic approaches for identifying metabolic dysfunction before the onset of overt clinical disease.

Alterations in branched-chain amino acid (BCAA) metabolism have been consistently associated with IR, obesity, and T2DM [22,23,29,30,31]. In the present study, increased urinary α-ketoisovalerate, a functional marker of altered L-valine catabolism and biotin-dependent carboxylase activity, suggests early impairment of oxidative BCAA pathways [32,33,34,35]. These findings are consistent with previous reports demonstrating that disruptions in BCAA metabolism precede overt hyperglycemia and are associated with lipid accumulation [32], mitochondrial inefficiency [36,37], and reduced metabolic flexibility [35,38]. Notably, these alterations were observed at insulin concentrations corresponding to the lower quartiles of conventional reference values, indicating that metabolic stress may occur before classical diagnostic thresholds are reached.

Similarly, increased urinary kynurenate excretion was associated with higher triglyceride-to-HDL cholesterol ratios and a markedly increased relative risk of IR. The kynurenine pathway is highly sensitive to inflammatory and metabolic stress, and its upregulation has been described in conditions characterized by chronic low-grade inflammation and increased cardiometabolic risk [39,40,41]. Our findings support the concept that disturbances in tryptophan metabolism can be identified early through the analysis of organic acidurias [20,38,42], providing insights into immunometabolic dysregulation prior to the clinical manifestation of disease and enabling opportunities for primary prevention.

The associations observed between increased adiposity, IR, and altered metabolite excretion reinforce the central role of insulin as a key regulator of lipid, carbohydrate, and amino acid metabolism [43,44,45]. In this cohort, the increase in fat mass was not accompanied by a proportional increase in fat-free mass, a major determinant of basal metabolic rate [46,47]. This imbalance may further contribute to metabolic inflexibility and disease progression by reducing energy expenditure and impairing substrate oxidation [32,48].

**Figure 5:**
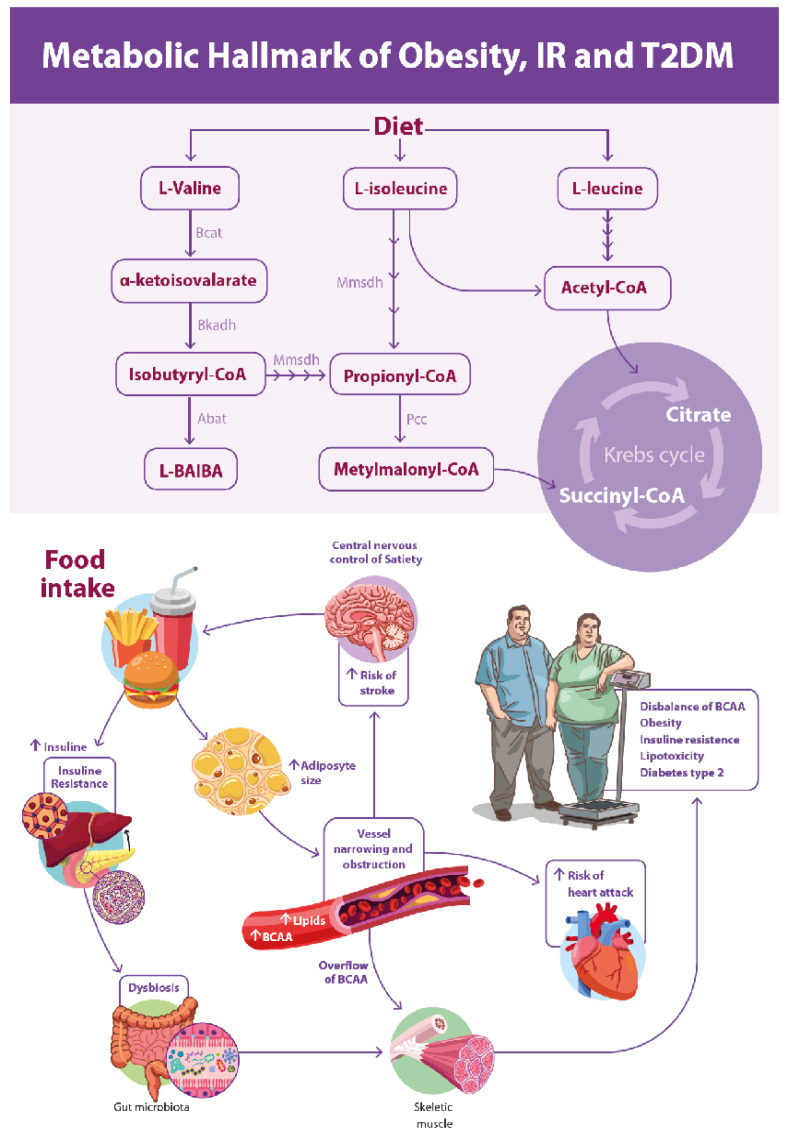
Proposed integrative model of metabolic hallmarks associated with obesity, insulin resistance, and type 2 diabetes mellitus. The upper panel illustrates dietary branched-chain amino acid (BCAA) metabolism, highlighting the catabolic pathways of L-valine, L-isoleucine, and L-leucine and their conversion into key intermediates, including α-ketoisovalerate, propionyl-CoA, methylmalonyl-CoA, succinyl-CoA, acetyl-CoA, and L-β-aminoisobutyric acid (L-BAIBA), which connect BCAA metabolism to the tricarboxylic acid (Krebs) cycle. The lower panel depicts the progressive development of obesity and its metabolic consequences, integrating increased food intake, gut dysbiosis, BCAA overflow, insulin resistance, adipocyte hypertrophy, lipotoxicity, and central nervous system dysregulation of satiety. These interconnected pathways contribute to the development of type 2 diabetes mellitus (T2DM) and increased cardiovascular and cerebrovascular risk.

Based on the integration of the present findings with existing literature, we propose an integrative model of immunometabolic dysregulation linking dietary intake, BCAA and tryptophan metabolism, adiposity, insulin resistance, and cardiometabolic risk, as summarized in Figure 5. This model highlights how early disturbances in amino acid catabolism and lipid handling may converge to promote metabolic stress long before overt clinical disease becomes apparent.

The strength of this study lies in the use of urinary organic acid analysis, which reflects metabolic activity over several hours, in contrast to plasma biomarkers that capture only a brief metabolic snapshot. This methodological approach may increase sensitivity for detecting early metabolic alterations. However, the study is limited by its convenience sample, relatively small subgroup sizes—particularly in sex-stratified analyses—and its cross-sectional design, which precludes causal inference and limits generalization.

Despite these limitations, the findings support the hypothesis that the urinary metabolomic profile of organic acids can identify early biochemical alterations associated with insulin resistance and cardiometabolic risk [39,42]. This approach may complement traditional biomarkers and contribute to primary prevention strategies aimed at preserving metabolic health and promoting healthy aging.

## 5. Conclusions

This study demonstrates that the urinary metabolomic profile can identify early metabolic alterations associated with insulin resistance in a cross-sectional clinical context, even in individuals whose conventional laboratory parameters remain within reference ranges. Elevated urinary α-ketoisovalerate and kynurenate were associated with higher fasting insulin levels and increased triglyceride-to-HDL cholesterol ratios, respectively, indicating that early disturbances in branched-chain amino acid and tryptophan metabolism may precede clinically evident alterations in insulin metabolism.

Although limited by sample size and study design, these findings support the use of urinary organic acid analysis as a complementary tool for the early identification of metabolic risk. This approach may facilitate timely preventive interventions aimed at preserving metabolic health, maintaining immunometabolic balance, and reducing the burden of chronic noncommunicable diseases.

## Figures and Tables

**Table 1:** Anthropometric parameters obtained by tetrapolar bioelectrical impedance analysis. Values are expressed as mean ± standard deviation (SD).

	Men (19)			Women (15)		
Parameter	Normal	Obese	Diabetic	Normal	Obese	Diabetic
Body weight (kg)	84 ± 15	114 ± 9	96.5 ± 23	58 ± 6 *	78 ± 5	85.5 ± 17
Adipose Mass (kg)	16 ± 4	34 ± 4 ^#^	36 ± 9 ^#^	24 ± 3 *	42 ± 2 *	40 ± 1

* *p* ≤ 0.05 indicates a statistically significant difference between men and women within the normal-weight and obese groups. ^#^ *p* ≤ 0.05 indicates a statistically significant difference in adipose mass between sexes in overweight and obese individuals, demonstrating increased adiposity. Statistical analyses were performed using two-way analysis of variance (ANOVA) with a completely randomized design.

## Data Availability

The data presented in this study are not publicly available due to ethical and privacy restrictions related to human participant data. Data may be made available from the corresponding author upon reasonable request and with approval from the institutional ethics committee.

## Abbreviations

The following abbreviations are used in this manuscript:

IR	Insulin Resistance
T2DM	Type 2 diabetes mellitus
NAFLD	Non-alcoholic fatty liver disease
CVD	Cardiovascular disease
TG	Triacylglycerol
HDL-C	High-density lipoprotein cholesterol
TRP	L-tryptophan
KP	Kynurenine pathway
KIN	Kynurenine
KYNA	Kynurenic acid
TDO	Tryptophan 2,3 dioxygenase
IDO	Indoleamine 2,3 dioxygenase
BCAA	Branched-chain amino acid
BCAT	Branched-chain amino acid transaminase
BCKDH	Branched-chain α-ketoacid dehydrogenase
RR	Relative Risk
ICF	Informed consent form
BMI	Body mass index
HOMA-IR	Homeostasis Model Assessment of Insulin Resistance
FI	Fasting insulin
FG	Fasting glucose
SAH	Systemic Arterial Hypertension
NIH	National Institutes of Health
LC-MS/MS	Liquid chromatography with tandem mass spectrometric detection
SD	Standard Deviation
SEM	Standard error of the mean
P	Percentiles
ANOVA	Analysis of variance
HbA1c	Glycated hemoglobin
L-BAIBA	L-β-aminoisobutyric acid
LDL	Low-density lipoprotein
